# Targeting Senescent Cells to Counter Aging and Aging-Related Conditions

**DOI:** 10.1093/geroni/igaa057.2981

**Published:** 2020-12-16

**Authors:** Nathan LeBrasseur

**Affiliations:** Mayo Clinic, Rochester, Minnesota, United States

## Abstract

In response to various forms of age-associated damage, cells can enter a state of senescence. Senescent cells can compromise the health and function of a tissue, and their accumulation with advancing age is believed to contribute to age-related diseases and geriatric syndromes. In preclinical models (i.e., mice), selective elimination of senescent cells through either genetic approaches or a new class of pharmacological agents, termed “senolytics”, has been show to effectively delay, prevent, or reverse the onset and/or progression of pulmonary disease, osteoporosis, atherosclerosis, diabetes, cognitive decline, and several other conditions. Thus, considerable efforts are underway to optimize pharmacological strategies and test their effectiveness in human populations. This seminar will highlight the state-of-the-science of senolytic drugs, and the opportunities and challenges for early phase clinical trials in humans.

